# Moderated serial mediation effects of adaptation problems, academic stress, and interpersonal relationships on the sleep quality of early-year university students

**DOI:** 10.3389/fpubh.2024.1476020

**Published:** 2024-11-13

**Authors:** Chenyang Zhao, Yang Zhang

**Affiliations:** ^1^College of Physical Education, Hunan Normal University, Changsha, China; ^2^Independent Researcher, Windermere, FL, United States

**Keywords:** anxiety, mental health, insomnia, Pittsburgh sleep quality index, social networking

## Abstract

**Purpose:**

Due to heightened anxiety levels, sleep disorders become more prevalent among university students. This study, centered on adaptive capacity in early-year university students, aimed to explore the underlying mechanisms through which anxiety affects sleep quality.

**Methods:**

Between February 2023 and June 2023, a cohort of first- and second-year university students (mean age 18.8 years) from Hunan Province, China, took part in an online survey. Anxiety was assessed using the Self-rating Anxiety Scale; adaptation problems, academic stress, and interpersonal relationships were assessed using the Adolescent Self-Rating Life Events Checklist; and sleep quality was assessed using the Pittsburgh Sleep Quality Index (PSQI). A moderated serial mediation model, based on Hayes’ Model 92, was used to assess the hypothesized model.

**Results:**

The survey resulted in 3,490 valid responses. Among respondents, 24% exhibited anxiety symptoms and 30.4% showed abnormal sleep patterns (i.e., PSQI score ≥ 8). The most significant path identified based on indirect effects was anxiety (*β* = 0.109), adaptation problems (*β* = 0.183), academic stress (*β* = 0.081), and sleep quality (all p-values < 0.001). Furthermore, interpersonal relationships moderated the effects of adaptation problems (*β* = −0.015, *p* < 0.001), academic stress (*β* = −0.012, *p* < 0.001), and anxiety (*β* = 0.003, *p* = 0.002) on sleep quality.

**Conclusion:**

Elevated anxiety and sleep disorders are common among university students. Adaptive capacity may play a crucial role in sleep disorders among early-year university students. Interventions such as fostering strong interpersonal relationships in campus environments, may prove beneficial in improving academic performance and sleep quality.

## 1 Introduction

It is well established that sleep disorders cause the development of noncommunicable diseases and mental illnesses (1), highlighting the crucial role of sleep in an individual’s well-being. However, insufficient sleep has evolved into a global public health concern. According to a global sleep survey, over 70% of respondents reported experiencing one or more new sleep disorders, with only 55% of adults expressing satisfaction with their sleep quality (2). While various factors contribute to poor sleep quality, anxiety stands out as a primary cause among young people (3). From the lens of biological evolution, anxiety tends to peak during young adulthood, driven by pressures related to reproduction and social selection. University students, transitioning into independent maturity, often face heightened anxiety levels (4, 5). In recent years, sleep disorders have become a prevalent issue among Chinese university students, impacting their academic performance and well-being (6). A national survey revealed that Chinese university students exhibit patterns of late sleep initiation (average = 23:28 h), prolonged sleep onset delay (average = 31 min), and sleep deprivation (average = 6.87 h/day) (7). Therefore, delving into the mechanisms underlying poor sleep quality among Chinese university students carries significant theoretical and practical importance.

## 2 Theoretical framework

Firstly, it is useful to differentiate between various types of sleep disorders. Sleep disorders encompass disruptions in an individual’s sleep–wake cycle, stemming from conditions such as insomnia, sleep apnea, irregular breathing during sleep, restless legs syndrome, narcolepsy, and non-24-h sleep–wake disorder. Insomnia, characterized by difficulty falling asleep, staying asleep, or waking too early, was traditionally more prevalent among older adults. However, epidemiological studies now indicate an increasing prevalence of this sleep disorder among young people across various socioeconomic conditions (8–11). Consequently, insomnia has become an increasingly public health concern among young people, prompting this study to focus on the poor sleep quality of university students attributed to insomnia.

**Figure 1:**
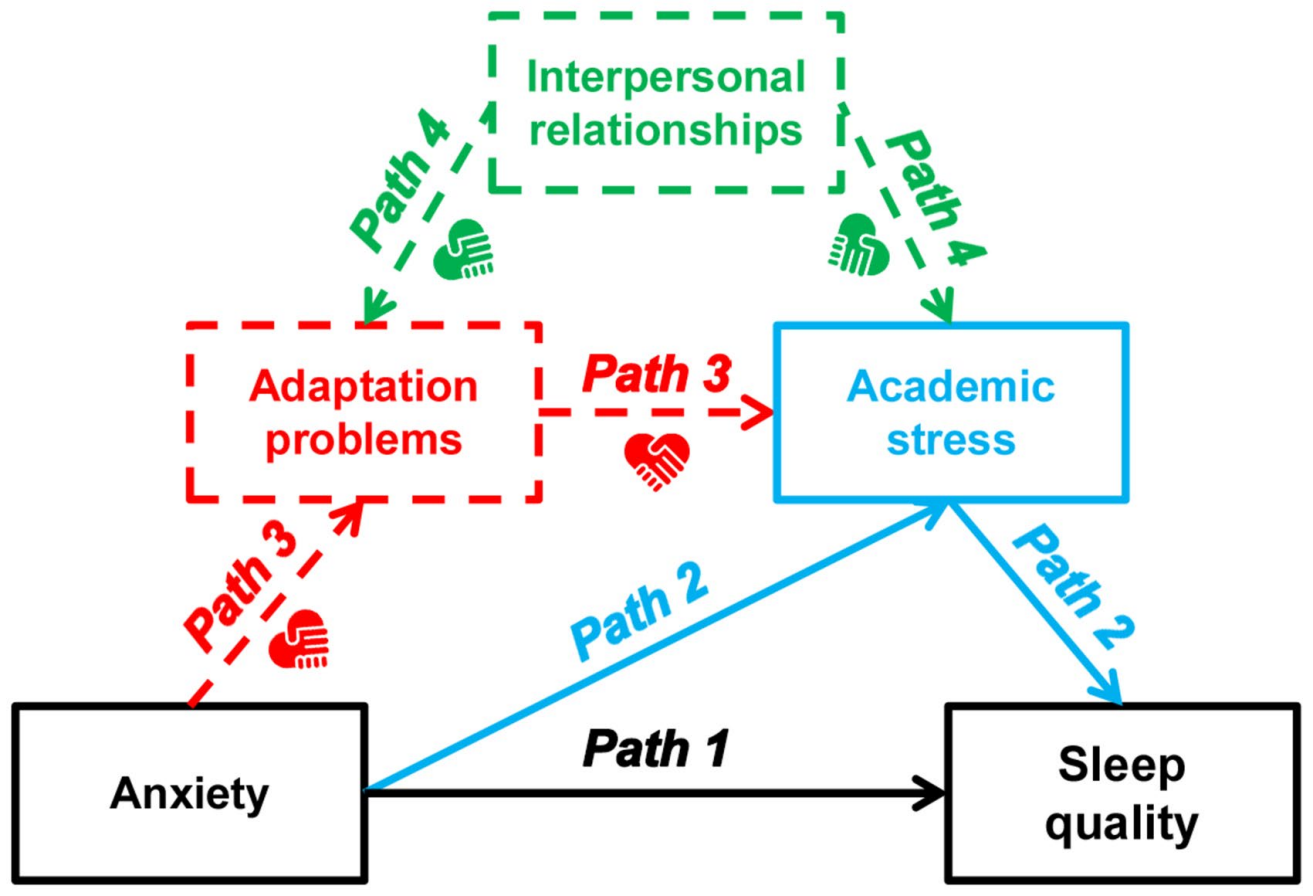
A hypothesized model between anxiety and sleep quality among early-year university students. This relationship is depicted through arrows indicating the directional influence of mediators and moderator on sleep quality. The solid lines represent the known relationships in the literature. The dashed lines signify areas where further investigation is needed. The heart/hand icon indicates potential opportunities for intervention if the hypothesized mediation path 3 and moderation path 4 could be validated.

Currently, two main theories explain the relationship between anxiety and sleep quality in school-aged youth, as illustrated in Figure 1 (see also paths 1 and 2). First, a strong relationship between anxiety and sleep quality has been well established (12), as indicated by path 1. University life presents numerous daily sources of anxiety. For instance, students from low- to middle-class families may struggle with feelings of loss stemming from unstable social status yet possess a strong desire to ascend the socioeconomic ladder (13). This leads to intense anxiety about an uncertain future and sometimes feelings of hopelessness. Similarly, social media has emerged as a new source of anxiety, often linked to comparisons of perceived life satisfaction (14). Regardless of the specific sources of anxiety, its accumulation can significantly impact mental health and contribute to poor sleep quality. Second, the most frequent periodic source of anxiety among university students is related to academic performance and the bidirectional relationship between academic stress and sleep quality has been extensively studied (15), as indicated by path 2. Freshmen often experience anxiety due to the transition in learning styles from high school to university, while senior students face pressure related to project completion and graduation. Academic stress acts as a negative mediator between daily anxiety and sleep quality (path 2 in Figure 1) throughout academic semesters. Furthermore, academic burnout resulting from poor test outcomes can transit a temporary sleep reduction into a chronic sleep disorder (16).

However, the proposed effects of anxiety and/or academic stress may be influenced by additional co-factors. Zhang and colleagues proposed that rumination and resilience serve as psychological traits mediating the link between stress and sleep quality among university students (17). In a broader context, their theory implies that an individual’s ability to adapt to adversity or new environments may impact how anxiety affects sleep quality. This is a crucial point in the Chinese context: the majority of Generation Z, comprising about 20% of the country’s population, were born into arguably the most rapid socioeconomic transition in modern Chinese society. China has been accelerating its urbanization process since 2014, which has brought about short-term challenges. The high cost of urban living not only poses resistance to urban modernization but also triggers various financial and social anxieties in youth (18). Integrating young individuals from low- and middle-income families into modern urban life necessitates an adaptation process. Uncertainty stress, stemming from these challenges, is known to impact one’s mental health (19) and sleep quality (20). This underscores the need for the new generation of Chinese youth to cultivate and demonstrate higher adaptive capacity to navigate these changes effectively. Additionally, university students studying away from their hometown are more susceptible to life event adjustments, leading to mood swings (21). Difficulties in adaptation may result in reduced learning capabilities, contributing to heightened academic stress. Accordingly, we propose the existence of an indirect effect as depicted in path 3 of Figure 1. The first hypothesis is stated as follows:

*H1*: Adaptation problems mediate the relationship between anxiety and academic stress, thereby impacting sleep quality among Chinese university students.

These interactions are further complicated by the existence of social networks. Interpersonal relationships are integral to social networks, and the behaviors of others, as well as the occurrence of negative events within these networks, can profoundly influence individuals. Given the centrality of interpersonal relationships in mental health theories (22, 23), it is plausible that interpersonal relationships could act as a moderating factor for adaptation problems and academic stress. Indeed, a few studies have supported this notion. For example, Zhang et al. found that interpersonal relationships could enhance school adaptation by providing increased social support and fostering resilience, which was correlated with improved adaptation (24). Similarly, Ye et al. identified disturbances in interpersonal relationships as risk factors for social adjustment (25). Additionally, Kiuru et al. demonstrated that high-quality interpersonal relationships were linked to higher academic achievement through improved school well-being (26). Hence, we propose the second hypothesis, depicted in path 4 of Figure 1, as follows:

*H2*: Interpersonal relationships may either positively or negatively moderate the relationship between anxiety and sleep quality, thereby affecting sleep quality among Chinese university students.

In summary, current gaps include the specific mechanisms through which anxiety affects sleep quality and the moderating factor that influences this relationship, and potential interventions to improve sleep quality in university students. Therefore, this study aimed to address a theoretical gap by proposing a moderated serial mediation model between anxiety and sleep quality. In this model, we hypothesized that adaptation problems and academic stress sequentially mediate these effects, with interpersonal relationships moderating their impact on sleep quality. Ultimately, this theoretical model could inform the development of targeted interventions to mitigate sleep disorders in university students, especially as they navigate critical transitions toward career advancement stages.

## 3 Methods

### 3.1 Participants

The study was conducted in accordance with the Declaration of Helsinki, and approved by the Ethics Committee of Hunan Normal University (protocol code 2023 No.362). This survey-based research invited first- and second-year students from Hunan Normal University, Central South University, Hunan University, Hunan Agricultural University, and Changsha University of Science and Technology to participate. The exclusion criteria include students currently undergoing mental health treatments or taking medications for sleep disorders. All participants or their legal guardians have provided written informed consent, which was electronically signed, for participation in this study.

### 3.2 Protocol

**Figure 2:**
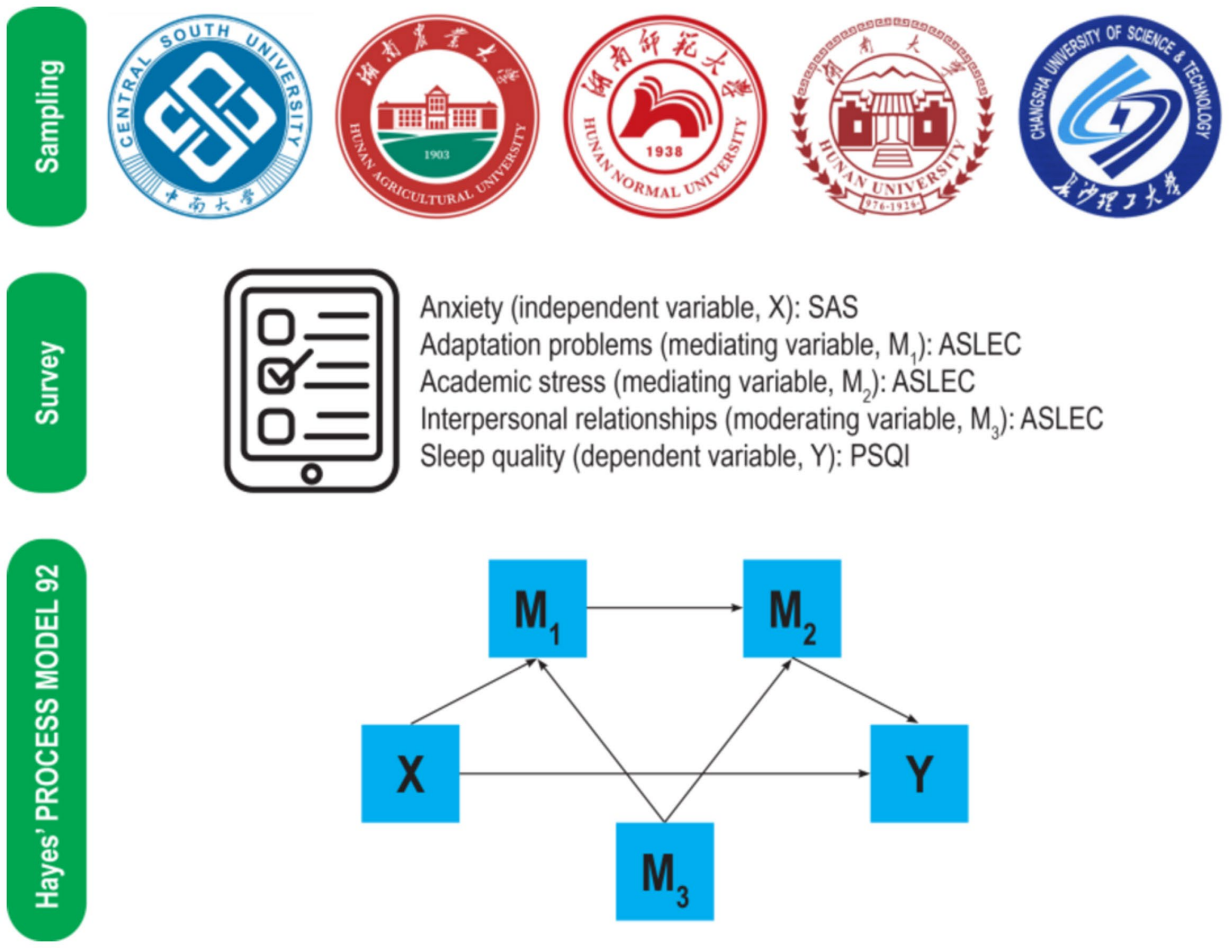
Flow chart of the study. ASLEC, Adolescent Self-Rating Life Events Checklist; PSQI, Pittsburgh Sleep Quality Index; SAS, Self-rating Anxiety Scale.

The data were collected using a survey method, employing a cluster random sampling approach. Through an online platform (Questionnaire Star, Changsha Ranxing Information Technology Co.), we distributed a total of 3,500 surveys to 700 students randomly selected from the aforementioned five universities between February 2023, and June 2023. Therefore, the survey was conducted during the second semester for first-year university students and the fourth semester for second-year university students. Figure 2 outlines the research process.

### 3.3 Instrument

Anxiety levels were assessed using the Self-rating Anxiety Scale (SAS) (27). The SAS has demonstrated satisfactory psychometric properties among Chinese university students (28), and Chinese-adapted norms were applied in this study for diagnosis. Scores of 70 or higher indicate severe anxiety, 60–69 indicate moderate anxiety, 50–59 indicate mild anxiety, and scores below 50 indicate normal states. In this study, Cronbach’s alpha and Kaiser-Meyer-Olkin (KMO) values were 0.74 and 0.90, respectively.

The Adolescent Self-Rating Life Events Checklist (ASLEC) (29) was used to evaluate the mediators and moderator in this study. The scale serves as a valuable tool for assessing mental health conditions and has been adopted in diverse populations (30, 31). The ASLEC comprises 26 items concerning negative life events, from which 11 items relevant to this study were selected. Factor 1 of the ASLEC pertains to interpersonal relationships, including “misunderstood/rejected by others” (item 1), “looked down upon” (item 2), “break up with close friends” (item 4), “cheated/ridiculed” (item 15), and “conflicts with family members” (item 25). Factor 2 of the ASLEC addresses academic stress and includes “failure in a test” (item 3), “homework overload” (item 9), “family financial problems” (item 16), “failure in a competition” (item 18), and “Entrance examination pressure” (item 22). Factor 4 of the ASLEC relates to adaptation problems, including “major change in life habits” (item 5), “living apart from family” (item 8), “personal serious illness/injury” (item 11), and “others” (item 27). Initially, respondents indicated whether each event occurred to them in the past 12 months, responding “yes” or “no.” If the event did not occur, it received a score of 0; otherwise, respondents rated the perceived stress of each experienced event on a five-point Likert scale ranging from 1 = “not at all” to 5 = “extremely severe.” The scores of individual items were summed to generate a factor score, with higher scores indicating a greater number of negative life events experienced in the past year. In this study, each factor’s Cronbach’s alpha was greater than 0.70, and the KMO value was 0.96.

Sleep quality was evaluated using the Pittsburgh Sleep Quality Index (PSQI). The higher the global PSQI score, the poorer the sleep quality. We applied Chinese-adapted norms for diagnosis (32), where a global PSQI score of eight or higher signifies abnormal sleep patterns. In this study, the Cronbach’s alpha and KMO values were 0.80 and 0.81, respectively.

### 3.4 Statistics

The data underwent preliminary analysis employing Student’s *t*-tests (33), one-way ANOVA with Fisher’s LSD *post hoc* test, Harman’s single-factor test, correlation analysis, and hierarchical multivariate regression, all conducted in IBM SPSS Statistics (version 24.0). The moderated serial mediation effects were estimated using Hayes’ PROCESS (version 3.5) Model 92. In conducting moderation analysis without prior knowledge about the population centiles, similar studies have utilized the mean score plus or minus one standard deviation to categorize groups (33). In this study, respondents were categorized into two groups based on scores in relation to the sample’s mean score. For example, those whose interpersonal relationships scores surpassed the mean (8.19) were placed in the above-average interpersonal relationships group, while those below the mean were placed in the below-average group. In this context, above-average scores indicate weaker interpersonal relationships. Hypothesized effects were evaluated using a bias-corrected non-parametric percentile bootstrap method with 5,000 random resamplings from the total sample size, and significance was determined at the 5% alpha level.

## 4 Results

### 4.1 Overview of data

A total of 3,490 valid questionnaires were collected. Table 1 presents the demographics of the respondents. With the exception of 19-year-old students, who exhibited significantly higher levels of anxiety and poorer sleep quality, no statistically significant differences were observed among the subgroups of interest. Of the survey respondents, 19.7% exhibited mild anxiety symptoms, 6% exhibited moderate anxiety symptoms, and 0.3% exhibited severe anxiety symptoms. Additionally, 30.4% of respondents had abnormal sleep patterns (i.e., PSQI score ≥ 8).

**Table 1:** Demographic characteristics.

Characteristic	*N*	*X*	*M*_1_	*M*_2_	*M*_3_	*Y*
Sex						
Male	1,557	39.4 ± 12.2	4.2 ± 2.5	8.1 ± 3.8	8.2 ± 4.5	6.3 ± 3.0
Female	1,933	38.7 ± 12.3	4.2 ± 2.6	8.1 ± 3.7	8.2 ± 4.3	6.3 ± 2.9
Age						
<18 years	204	37.5 ± 11.5*	4.1 ± 2.5	7.8 ± 3.6	7.9 ± 4.6	6.0 ± 2.8*
18 years	1,222	38.4 ± 12.1*	4.2 ± 2.5	8.0 ± 3.7	8.1 ± 4.3	6.2 ± 2.8*
19 years	1,334	39.7 ± 12.5	4.2 ± 2.6	8.2 ± 3.7	8.4 ± 4.4	6.5 ± 3.0
>19 years	730	39.3 ± 12.0	4.3 ± 2.7	8.3 ± 3.9	8.0 ± 4.6	6.4 ± 3.2
Residence						
Rural	1,971	38.9 ± 12.1	4.2 ± 2.5	8.1 ± 3.7	8.2 ± 4.3	6.2 ± 2.9
Urban	1,519	39.3 ± 12.4	4.3 ± 2.6	8.1 ± 3.7	8.2 ± 4.5	6.5 ± 3.0
Ethnicity						
Han	2,792	38.8 ± 12.2	4.2 ± 2.6	8.0 ± 3.7	8.1 ± 4.4	6.3 ± 3.0
Minority	698	39.9 ± 12.5	4.3 ± 2.5	8.4 ± 3.8	8.5 ± 4.4	6.4 ± 3.0
Year of study						
First year	1,642	38.3 ± 12.1	4.2 ± 2.5	8.0 ± 3.7	8.1 ± 4.3	6.2 ± 2.8
Second year	1,848	39.7 ± 12.5	4.2 ± 2.6	8.2 ± 3.7	8.4 ± 4.4	6.5 ± 3.0
Overall	3,490	39.1 ± 12.2	4.2 ± 2.5	8.1 ± 3.7	8.2 ± 4.4	6.3 ± 3.0

Data are expressed as mean and standard deviation. *X*, anxiety; *M*_1_, adaptation problems; *M*_2_, academic stress; *M*_3_, interpersonal relationships; *Y*, sleep quality. * denotes *p* < 0.05 vs. 19 years old.

### 4.2 Common method bias

Harman’s single-factor test was used to assess common method bias. The unrotated factor analysis revealed a total of 16 common factors with eigenvalues exceeding 1. The first factor explained 16.18% of the total variance, falling below the threshold of 40%. This result suggests no common method bias in the survey.

### 4.3 Multivariate regression

Table 2 shows the partial correlations between all variables, which are significantly positive. Hierarchical multiple linear regression (Table 3) shows that anxiety was a significant predictor of sleep quality. When adaptation problems and academic stress were incorporated into the regression model (step 4), all these variables were significant predictors of sleep quality.

**Table 2:** Partial correlation analysis.

	1	2	3	4	5
Interpersonal relationships	1	–	–	–	–
Adaptation problems	0.39***	1	–	–	–
Academic stress	0.49***	0.44***	1	–	–
Anxiety	0.59***	0.57***	0.60***	1	–
Sleep quality	0.36***	0.35***	0.37***	0.46***	1

***denotes *p* < 0.001.

**Table 3:** Hierarchical multivariate regression.

Step	Predictors	Outcome	*R* ^2^	*F*	*β*	*t*	*p*	VIF
1	Anxiety	Sleep quality	0.216	963	0.465	31.0	<0.001	1.0
2	Anxiety	Adaptation problems	0.324	1,673	0.569	40.9	<0.001	1.0
3	Anxiety	Academic stress	0.377	1,057	0.516	31.8	<0.001	1.5
	Adaptation problems		–	–	0.150	9.2	<0.001	1.5
4	Anxiety	Sleep quality	0.237	361	0.329	16.1	<0.001	1.9
	Adaptation problems		–	–	0.112	6.1	<0.001	1.5
	Academic stress		–	–	0.121	6.4	<0.001	1.6

VIF, variance inflation factor.

### 4.4 Structured model

**Figure 3:**
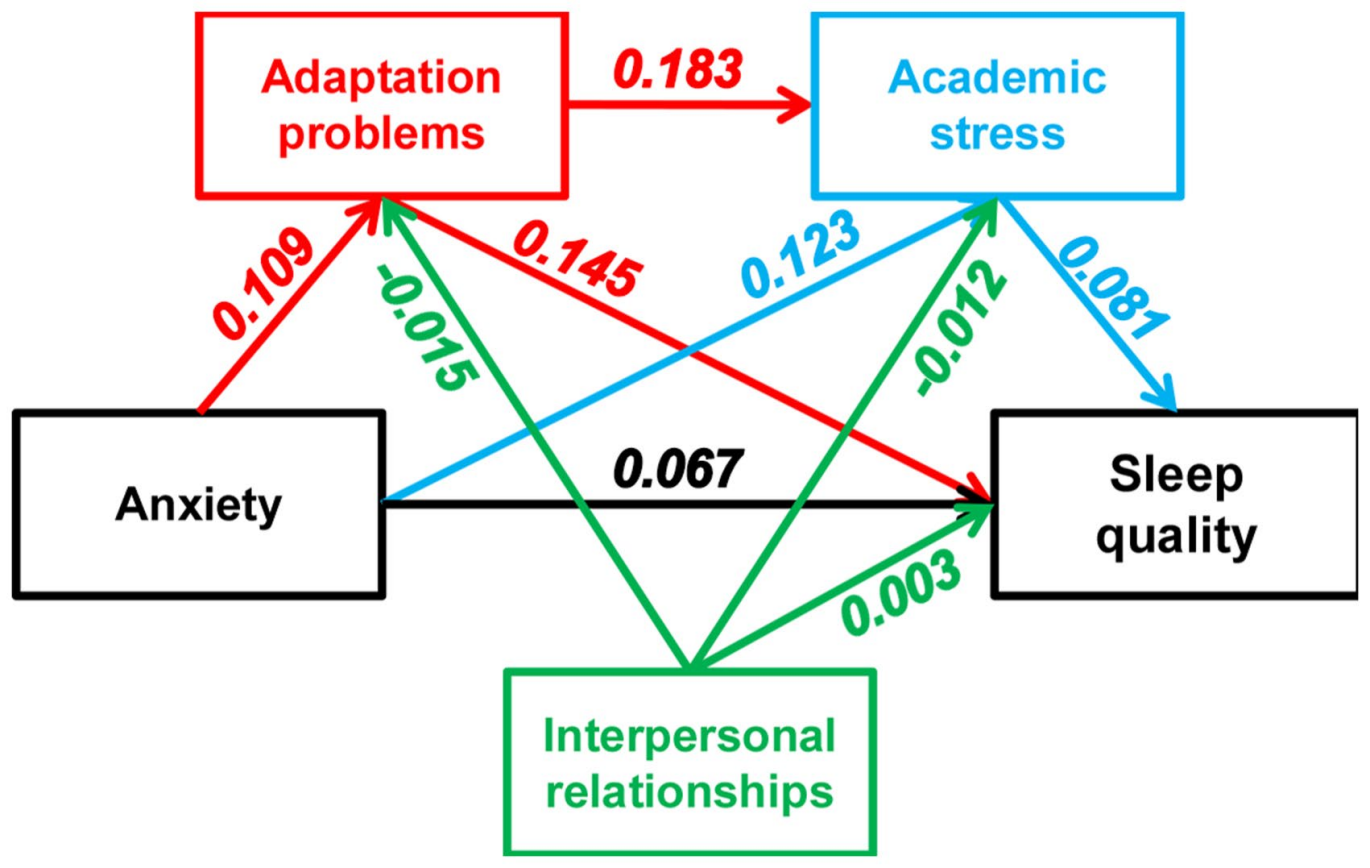
Conceptual diagram of moderated serial mediation model using Hayes’ PROCESS (Model 92). Beta values (all *p*-values < 0.01) represent indirect effects.

Table 4 provides a summary of the mediating and moderating effects observed. All mediation paths demonstrate significance, indicating a serial mediation effect, where adaptation problems and academic stress mediate the impact of anxiety on sleep quality among university students, with adaptation problems exhibiting the most pronounced indirect effect (*β* = 0.145). These findings are visually represented in Figure 3.

**Table 4:** Indirect effects of moderated serial mediation models.

Variable	Adaptation problems			Academic stress			Sleep quality		
	β	SE	*p*	β	SE	*p*	β	SE	*p*
Constant	−0.006	0.042	0.881	−0.040	0.057	0.483	6.387	0.052	<0.001
Interpersonal relationships	0.052	0.010	<0.001	0.169	0.014	<0.001	0.070	0.013	<0.001
Anxiety	0.109	0.004	<0.001	0.123	0.006	<0.001	0.067	0.005	<0.001
Adaption problems	–	–	–	0.183	0.024	<0.001	0.145	0.022	<0.001
Academic stress	–	–	–	–	–	–	0.081	0.015	<0.001
int_1	0.000	0.001	0.773	0.000	0.001	0.798	0.003	0.001	0.002
int_2	–	–	–	0.007	0.005	0.149	−0.015	0.004	<0.001
int_3	–	–	–	–	–	–	−0.012	0.003	<0.001
*F*	570.3			471.8			165.3		
*R* ^2^	0.329			0.404			0.249		

int_1 = anxiety × interpersonal relationships; int_2 = adaptation problems × interpersonal relationships; int_3 = academic stress × interpersonal relationships.

Meanwhile, three significant moderating paths emerged, the effects of which are presented in Table 5. First, interpersonal relationships negatively moderated the impact of anxiety on sleep quality. When anxiety was absent, university students with above-average scores in interpersonal relationships experienced poorer sleep quality compared to those with below-average scores in interpersonal relationships. However, when respondents exhibited anxiety symptoms, interpersonal relationships did not exert further influence on sleep quality. The second significant path identified was the moderating role of interpersonal relationships in the mediation model linking anxiety to sleep quality through adaptation problems. It was observed that university students with above-average scores in interpersonal relationships reported poorer sleep quality, regardless of their adaptation problems. Finally, in cases where anxiety influences sleep quality through both the adaptation problems and academic stress factors, it was found that university students with stronger interpersonal relationships, regardless of their academic stress levels, experienced better sleep quality than their peers with weaker interpersonal relationships.

**Table 5:** The moderating effects of interpersonal relationships on sleep quality of university students.

	Interpersonal relationships	
	Below-average group	Above-average group
Anxiety		
No anxiety group	5.2 ± 2.6	6.5 ± 2.3***
Mild anxiety group	8.0 ± 3.2	8.0 ± 2.7
Moderate anxiety group	9.7 ± 3.2	9.3 ± 3.4
Adaptation problems		
Below-average group	5.1 ± 2.7	6.6 ± 2.5***
Above-average group	6.6 ± 2.9	8.0 ± 2.9***
Academic stress		
Below-average group	5.1 ± 2.6	6.7 ± 2.7***
Above-average group	6.6 ± 3.0	7.8 ± 2.8***

Data (Pittsburgh Sleep Quality Index score total score) are expressed as mean and standard deviation. The cutoff criteria applied in grouping were determined based on the following scores: mean score of adaptation problems = 4.22; mean score of academic stress = 8.11; mean score of interpersonal relationships = 8.19. *** denotes *p* < 0.001 vs. below-average group.

## 5 Discussion

According to a recent review of epidemiological studies in China, the pooled PSQI total score was 4.32 (95% confidence interval 3.82–4.81), with one in five individuals in the general Chinese population experiencing poor sleep quality (34). In our study, 30.4% of survey respondents reported abnormal sleep patterns, with a mean PSQI score of 6.33 (95% confidence interval 6.23–6.41). It is worth noting that if this study had employed an international cutoff criterion such as a PSQI total score of ≥6 (35), 59.3% of this sample may have been categorized as presenting sleep disorders. These data highlight the prevalence of sleep disorders among early-year university students from Changsha, China. In line with existing literature (36, 37), our findings suggest that anxiety can negatively predict sleep quality, with academic stress acting as a mediator between these two variables. This study contributes to our understanding of the underlying mechanisms linking anxiety, academic stress, and sleep quality by introducing two new theoretical viewpoints.

First and foremost, adaptation problems not only acted as a partial mediator (total effect *β* = 0.321: anxiety → adaptation problems → sleep quality) but also, more importantly, served as a serial mediator (total effect *β* = 0.440: anxiety → adaptation problems → academic stress → sleep quality) between anxiety and sleep quality. To our knowledge, this pathway has not been previously reported in the literature on Chinese university students. In Chinese culture, pre-university education is rigorous, with adolescents spending the majority of their time in high school engaged in curriculum activities. The transition from high school, characterized by semi-open management and strong family support, to independent, open management universities, brings about significant changes in the social environments surrounding university students. This transition often triggers high levels of anxiety, which can lead to various mental health issues. For instance, the open nature of campus environments provides numerous opportunities for socializing with new peers, making friends, and dating. However, data suggest that social life can bring as much joy as it can depression and anxiety in young adults (38, 39). For early-year university students who have not yet developed communication skills and adaptive skills for independent living, heightened anxiety could lead to adaptation problems (40). These problems, in turn, may lead to other unhealthy behaviors (41, 42), academic stress, and ultimately influence sleep quality. Furthermore, the rapid transition in roles is exemplified by an unfamiliar living environment. In a study involving Tibetan university students studying outside of Tibet, Kang et al. found that 45.3% of this cohort presented mild mental health issues (43), indicating the impact of relocation on university life. Our finding suggests that adaptive capacity is a critical factor in determining the academic performance and sleep quality of early-year university students.

Second, interpersonal relationships may act as both a remedy and a catalyst for poor sleep quality. In this study, interpersonal relationships partially moderated the effects of adaptation problems (*β* = −0.015), academic stress (*β* = −0.012), and anxiety (*β* = 0.003) on sleep quality. Respondents with below-average scores in interpersonal relationships had better sleep quality compared to those with above-average scores, regardless of their adaptation problems. This suggests that strong interpersonal relationships can mitigate the negative impact of adaptation problems on sleep quality. Zhang et al. also reported that interpersonal relationships can enhance social support, thereby alleviating adaptation problems (24). Our findings not only corroborate Zhang et al.’s conclusion but also extend the effect to sleep quality. Regarding academic stress, respondents with below-average scores in interpersonal relationship slept better than those with above-average scores, irrespective of their level of academic stress. This finding aligns with existing research on the effect of interpersonal relationships on academic performance (26) and broadens our understanding of its moderating role in sleep quality. However, effectively managing interpersonal relationships could sometimes deplete emotional resources (44), leading to a negative feedback loop known as “I am tired” (45), characterized by a high demand for interpersonal relationships, depleted emotional resources, and consequently heightened anxiety. In a study of first-year Chinese university students, Deng et al. found that extraversion displayed a curvilinear relationship with depression (39). Specifically, the relationship was significantly negative from lower to moderate levels of extraversion, but it plateaued at higher levels. Essentially, beyond a certain threshold, actively maintaining positive interpersonal relationships may not provide additional benefits for improving mental health. This phenomenon was also observed in our study: as anxiety levels increased, respondents with below-average scores in interpersonal relationship exhibited deteriorating sleep quality. This suggests that while maintaining strong interpersonal relationships within normal anxiety levels can positively affect sleep quality, unchecked anxiety may diminish the beneficial effects of interpersonal relationships.

From a practical standpoint, future research could intervene in the pathway of anxiety’s impact on sleep quality by focusing on the pivotal mediating factor identified in this study: adaptability. While empirical studies suggest that the adaptive capacity of university students can be enhanced through support in family relationships (46), we believe this approach may not offer a fundamental solution. As the saying attributed to Han Feizi goes, “distant water cannot put out a nearby fire.” The transition to university represents a major life transition: Campus life is expected to broaden students’ social networks, from which they begin to rely less on family and seek mutual support from peers. Accordingly, it is our opinion that the fundamental approach lies in developing the adaptive capacity of developing new social networks. Research has shown that university students who actively engage in university clubs tend to be better learners (47, 48), possibly due to the cultivation of positive interpersonal relationships that enhance adaptive capacity, subsequently facilitating learning. Likewise, some studies indicate that university students who participate actively in club activities experience better sleep quality (49, 50), which may also be influenced by the aforementioned mechanism. Therefore, participating in leisure-time social activities such as hometown associations, special interest groups, and professional readiness clubs may prove effective in assisting early-year university students in adapting to their new roles, environments, and communication skills, consequently improving academic performance and sleep quality. We propose that future research focuses on examining the effect of social activities on university students’ sleep quality.

This study has several limitations that warrant consideration. First, it relies on participants’ self-reported data, which may introduce response bias. For example, relying on self-ratings to assess interpersonal relationships may not be reliable, and future research could benefit from using peer ratings for a more accurate evaluation. Additionally, being a cross-sectional survey, this study precludes drawing causal conclusions. Second, the sample was drawn exclusively from Changsha, potentially limiting the generalization of the findings. Moreover, the mechanisms underlying sleep disorders among senior university students and graduate students may be influenced by other factors (37). Third, despite interpersonal relationships being a significant moderator, its effect (i.e., *β*) appears relatively minor. In this regard, the lack of a centile distribution of interpersonal relationships among university students hampers accurate grouping and may not reflect the true impact of strong/weak interpersonal relationships on the factors under examination.

In conclusion, this study established a serial mediation model with a moderating effect. Among the paths examined, the most significant one identified was anxiety leading to adaptation problems, subsequently influencing academic stress and sleep quality. While this pathway may not universally apply to all cultural contexts, it does shed light on addressing poor sleep quality among early-year Chinese university students, particularly by addressing adaptation challenges associated with transitioning to campus life. Essentially, these findings underscore the importance of young adults developing adaptive capacity and interpersonal relationships to navigate various challenges in a highly competitive society.

## Data Availability

The raw data supporting the conclusions of this article will be made available by the authors, without undue reservation.
